# Negative social media-related experiences and lower general self-efficacy are associated with depressive symptoms in adolescents

**DOI:** 10.3389/fpubh.2022.1037375

**Published:** 2023-01-06

**Authors:** Tore Bonsaksen, Anne Mari Steigen, Tonje Holte Stea, Annette Løvheim Kleppang, Lars Lien, Marja Leonhardt

**Affiliations:** ^1^Department of Health and Nursing Sciences, Inland Norway University of Applied Sciences, Elverum, Norway; ^2^Department of Health, VID Specialized University, Stavanger, Norway; ^3^Department of Health and Nursing Science, University of Agder, Kristiansand, Norway; ^4^Department of Public Health and Sports Sciences, Inland Norway University of Applied Sciences, Elverum, Norway; ^5^Norwegian National Advisory Unit on Concurrent Substance Abuse and Mental Health Disorders, Innlandet Hospital Trust, Brumunddal, Norway; ^6^Faculty of Health Studies, VID Specialized University, Oslo, Norway

**Keywords:** adolescents, depressive symptoms, general self-efficacy, nationwide study, social media

## Abstract

Social media are an integral part of adolescents' daily lives, and reviews have suggested an overall small association between more social media use and mental health problems. However, researchers have commonly investigated social media use in a time use perspective, rendering nuances in adolescents' social media experience less well explored. Thus, studies of relationships between social media use and mental health problems need also examine the nature of the events taking place on social media. This study aimed to examine depressive symptoms in adolescents in relationship to time spent on social media, negative social media-related experiences, and general self-efficacy. Data collected in a national survey, Ungdata 2021 (*n* = 139,841), was used. Multivariate linear regression analyses showed that time spent on social media was associated with depressive symptoms (β = 0.09, *p* < 0.001). However, negative social media-related experiences were more strongly associated with depressive symptoms (β ranging 0.09–0.22, all *p* < 0.001), and their inclusion weakened the initial association between time on social media and depressive symptoms. General self-efficacy was directly associated with lower symptom levels (β = −0.29, *p* < 0.001) but did not change the associations between social media use and depressive symptoms. The findings imply that not only time spent on social media, but in particular negative social media-related experiences, are related to depressive symptoms in Norwegian adolescents. General self-efficacy is an important resource for adolescents' mental health.

## Introduction

Social media broadly refers to applications that allow users to engage in virtual interactions, with wider or narrower audiences (1). Social media have become a prominent aspect of 21^st^ century culture, and they are widely adopted across the world (2, 3). Young people use social media more often than older people (4, 5), and certain types of image-based social media, such as Instagram and Snapchat, are more often preferred among people in the younger age groups (5). Most social media are financed through advertising that is algorithmically controlled, which means that each user gets content that is tailored to their special target group, and what the user has shown interest in in the past (6).

In Norway, 99% of young people between 13 and 18 years use social media, with YouTube, Snapchat, Instagram, Facebook, and TikTok being the most popular (7). Social media is an important arena for socialization among young people, and engaging with social media gives rise to positive as well as negative experiences (8). Compared to boys, girls appear to have more positive (e.g., communication, positive attention) and negative experiences (e.g., pressure, social comparison) on social media (8), and more negative experiences have also been found among adolescents with lower sociodemographic status (9). While longitudinal studies are still relatively few, studies of adolescents in Norway have found that more time spent on social media has predicted subsequent increases in alcohol use (10, 11) as well as increases in depressive symptoms and conduct problems (11). International studies have reported higher social media use to be associated with more conflict within the family (12) and lower satisfaction with family life (13). Together, the studies indicate a need for further research on the possible long-term consequences of social media use among adolescents, as well as on consequences for the families involved.

There is a need to expand the scope of inquiry beyond the mere time spent on social media to also include the nature of the interactions taking place on social media and the consequences they may have. While some have emphasized social media's positive potential for connection and inclusion (14–16), others have argued that social media are substitutes for real-life relationships and rather serve to increase stress, such as “fear of missing out” (17, 18). For adolescents, social media use may instigate a pressure toward having a large number of followers and receiving affirmation (“likes”) as indicators of social acceptance, and lack of affirmation has been found to result in negative emotions and higher levels of depressive symptoms (19). In a large cross-national study of adolescents aged 11–15 years from 42 countries throughout Europe (weighted *n* = 180,919), about 12% have experienced cyber-bullying on social media, and more time spent on social media has been related to a greater risk of being bullied (20). Social media may in the case of late night use cause sleep problems (21, 22), which is a risk factor for attention problems in school (23) and for developing depression (24). However, such specific consequences of social media use may go unnoticed in studies where time use is the sole measure of social media use. Thus, when specific negative social media-related events are not considered, the commonly detected associations between time spent on social media and poorer mental health may concern very different circumstances and obscure more relevant relationships (25, 26).

Researchers have found diverging results with regard to the relationships between social media use and aspects of mental health. Whereas several studies have shown associations between higher levels of social media use and poorer mental health (18, 22, 27–29), recent reviews have indicated mixed results and an overall weak negative association across studies (1, 30). The great variety of methods for measuring both social media use and mental health outcomes may partly account for the mixed results (31). Associations between social media use and mental health have also been found to vary by gender, with more social media use related to poorer mental health in females (32). Thus, to move research forward, future studies should carefully specify the population of interest and which aspect of social media use is of interest.

Among young people, psychological resource factors such as general self-efficacy have been associated with favorable mental health outcomes in several studies over the last decades (33–35). In addition, self-efficacy has been found to moderate associations between various aspects of social media use and mental health, indicating that associations between more social media use and poorer mental health may be stronger for people with low self-efficacy (36, 37). Thus, while self-efficacy in adolescents may be vulnerable to the influence from social media, self-efficacy may also be viewed as a psychological resource that can buffer against a potentially negative influence from social media on mental health. The aim of the study was to examine depressive symptoms in adolescents in relationship to social media use, negative social media-related experiences, and general self-efficacy.

## Methods

### Design and procedure

A comprehensive cross-sectional study, the Ungdata survey, is offered annually to municipalities throughout Norway. Usually, the survey is conducted every third year in each municipality (38). In 2021 the survey was completed by 139,841 Norwegian adolescents, aged 13–19 years, representing 209 out of 356 Norwegian municipalities. Ungdata has become an essential source of information on young peoples' health and wellbeing, both at the national and municipal levels (see www.ungdata.no). The survey is conducted by the Norwegian Social Research (NOVA) at Oslo Metropolitan University in a collaboration with the Regional Drug and Alcohol Competence Centers (KoRus). The surveys are financed partially by the Norwegian Directorate of Health and gauge different aspects of adolescents' lives, i.e., health issues, local environment, school issues, social media use, diet, alcohol consumption, relationships with friends and parents, leisure time activities and symptoms of depression.

Parents and students were informed *via* mail in advance, and parents of under-age adolescents (13–17 years of age) were assured that they could withdraw their children from participation at any time. If no prohibiting response was received from parents of under-age adolescents, the adolescents decided independently whether or not to participate in the survey. They decided in school whether they wanted to participate after being informed that participation was voluntary and that they could skip questions that they did not want to answer. The study was conducted as a web-based questionnaire administered at school during school hours with a teacher or an administrator present to answer questions. The students used approximately 30–45 min to complete the questionnaire.

Some parts of the questionnaire are used nationally in all participating municipalities, whereas other parts are selected or de-selected by each municipality. Therefore, the number of responses vary greatly between mandatory and elective parts of the questionnaire. In this study, most employed variables were derived from the mandatory questions used nationally, while some variables (general self-efficacy, reports on conflicts or arguments in the family due to social media use, and reports on not getting enough sleep due to social media use) were derived from elective parts of the survey. In addition, it was possible to complete only parts of the survey and leave the remaining questions unanswered. Thus, the number of responses on a specific question varied according to (i) whether the question was included in the survey presented to the individual participants (i.e., a decision made by the municipality), and (ii) whether the individual participant chose to respond to it.

### Measures

#### Depressive symptoms

Depressive symptoms were measured using a six-item scale derived from the Depressive Mood Inventory (39), which was again based on the Hopkins Symptom Checklist (40). The adolescents were asked if during the past week they have been affected by any of the following: “Felt that everything is a struggle” (item 1), “had sleep problems” (item 2), “felt unhappy, sad or depressed” (item 3), “felt hopelessness about the future” (item 4), “felt stiff or tense” (item 5), “worried too much about things” (item 6). The six questions have four response categories: “Not been affected at all” (1), “not been affected much” (2), “been affected quite a lot” (3) and “been affected a great deal” (4). Sum scores were computed, ranging from 6 to 24; high scores indicated higher symptoms of depression. The depression scale has been psychometric evaluated among Norwegian adolescents. The scale showed good reliability (Person Separation Index: 0.802), and as a whole, the scale works reasonably well at a general level (41).

#### Sociodemographic factors

Gender (male/female), grade and centrality were used as covariates. Grade was used as an indicator for age, as age is not assessed within the survey. Centrality, as defined by Statistics Norway (42), was applied as a proxy for the grade of how centrally located a municipality in Norway is. Parents' level of education was measured by asking: “Did your parents go to university or to a university college?” Adolescents who were not in touch with one or both parents were asked to miss the question out. The response options were “No, none of them” (0) “Yes, one of them” (1), and “Yes, both of them” (2).

#### Social media use

Daily time spent on social media was measured by asking: “Think about what you do in a normal day, how much time do you spend on the following things: social media (Facebook, Instagram etc.).” Response options were no time, <30 min, 30 min−1 h, 1–2 h, 2–3 h and >3 h.

#### Negative social media-related experiences

Online bullying was assessed with the question “Have you been threatened or excluded on the internet?” with response options “yes, several times a week”; “yes, approximately once a week”; “yes, approximately once every 14 days”; “yes, approximately once a month”; “almost never” and “never.” Participants were asked whether the use of social media had resulted in “conflicts or arguments in the family” or “not getting enough sleep,” with response options “yes” and “no.” Further, the adolescents were asked to indicate on a five-point scale (not at all–very much) whether they had experienced “pressure to have many followers and likes on social media.” Responses on the scaled variables (“online bullying” and “pressure to have many followers and likes”) were recoded into dichotomous variables prior to analysis, with “never experienced online bullying” and “no pressure to have many likes and followers” coded as “no” and all other responses coded as “yes.”

#### Psychological resources

The *General Self-Efficacy Scale (GSE)* (43) is a questionnaire for assessing general self-efficacy among the general population aged 12 years and older. It has been translated into several languages (44), including Norwegian (45). The scale measures optimistic self-beliefs in coping with the demands of life. The short version of the GSE used in this study consists of 5 statements that respondents rate on a scale from 1 “completely disagree” to 4 “completely agree.” Example statements are: “I always manage to solve difficult problems if I try hard enough” (item 1) and “If someone opposes me, I can find the means and ways to get what I want” (item 2). The score is calculated by summing the individual's scores on all items. The score range is 5–20, with higher scores indicating higher self-efficacy. High correlations with self-appraisal, self-acceptance, and optimism indicate theoretical accuracy of the self-efficacy concept (46), and psychometric analyses of the GSE has consistently produced one latent dimension and high measures of internal consistency between items (47–49). In the current study, Cronbach's α was 0.87.

### Statistical analysis

Individuals who did not respond to specific items were removed casewise. The internal consistency of items in utilized scales were calculated by Cronbach's α. Descriptive analyses were performed for all employed measures. The dependent variable (depressive symptoms score) had a distribution which deviated from the normal distribution (Kolmogorov-Smirnov test *p* < 0.001), and it was skewed toward lower scores (skewness = 0.46, SE = 0.01) with the median value (Md = 12.0) being lower than the mean value (M = 12.9). However, skewed distributions frequently occur in large public health datasets without normally compromising the validity of parametric statistical testing (50). Therefore, we proceeded with parametric tests.

Depending on the number of groups to be compared, group differences in depressive symptoms were examined with independent *t*-tests or one-way ANOVA. In multiple comparisons, the Bonferroni correction was applied. Single (Model 0) and multiple linear regression analyses (Models 1–3) were used to assess associations between independent variables and depressive symptoms. In the multiple linear regression analyses, independent variables were entered in this sequence: Model (1) included daily time spent on social media and negative social media-related experiences (i.e., victim of online bullying; experienced conflicts or arguments in the family; experienced too little sleep due to social media use; and experienced pressure to have followers and likes on social media). Model (2) included the Model (1) variables while also including general self-efficacy. In Model 3, we included the Model (2) variables while adjusting for sociodemographic variables (age, gender, centrality, parents' education). Interaction analyses were performed to examine whether associations between negative social media-related experiences and depressive symptoms varied by gender and levels of general self-efficacy. In the event of statistically significant interactions, *post-hoc* analyses would repeat Model 3 separately for boys and girls and for participants with higher/lower general self-efficacy, respectively. Due to the large sample size, results with a corresponding *p* < 0.01 were considered statistically significant. The strength of associations (effect sizes) was interpreted in line with Cohen: β about 0.10 is small, about 0.30 is medium, and about 0.50 is large (51). Multicollinearity was checked with standard guidelines, stating that VIF should be below 5 and tolerance above 0.2 (52).

### Ethics

The study was conducted in accordance with the Declaration of Helsinki. Before participating in the study, all students were asked to provide informed consent. The parents received oral and written information about the study and were given the opportunity to withdraw their children from participation. The information letter was approved by The Norwegian Center for Research Data (NSD). All data were collected anonymously and then analyzed by independent researchers who did not participate in the collection of the data. The study was approved by the Institutional Ethics Review Board at Inland Norway University of Applied Sciences (protocol code 21/01894).

## Results

### Sample characteristics

The characteristics of the sample are shown in Table 1. Participants in the survey were 139,841 pupils from lower secondary (13–15 years of age) and upper secondary schools (16–19 years of age). The response rate in lower secondary schools was 83% and in upper secondary schools 67%, yielding an average response rate of 75% (38). The age distribution in the sample was similar between most categories, yet with declining participation rates in the higher age groups. The proportions of boys and girls were similar. The largest groups of participants lived in places classified to be in the middle range on the centrality index. Among 47% of the sample, both parents had higher education.

**Table 1:** Sample characteristics.

**Characteristics**	***n* = 139,841**
**Age cohort**	**n (%)**
8^th^ grade secondary school	27,078 (19.4)
9^th^ grade secondary school	26,667 (19.1)
10^th^ grade secondary school	26,243 (18.8)
1^st^ grade high school	23,021 (16.5)
2^nd^ grade high school	19,396 (13.9)
3^rd^ grade high school	13,054 (9.3)
**Gender**	**n (%)**
Boys	68,027 (48.6)
Girls	68,527 (49.0)
**Centrality index**	**n (%)**
1 (most central)	19,235 (13.8)
2	29,574 (21.1)
3	37,490 (26.8)
4	29,208 (20.9)
5	18,399 (13.2)
6 (least central)	5,840 (4.2)
**Parents with higher education**	**n (%)**
No parents with higher education	20,103 (14.4)
One parent with higher education	40,828 (29.2)
Both parents with higher education	66,309 (47.4)

Missing values represented 3.1% for age, 2.4% for gender, 0.1% for centrality, and 9.0% for parents with higher education.

### Depressive symptoms, general self-efficacy, social media use and negative social media-related experiences

Information about depressive symptom levels, general self-efficacy, social media use, and negative social media-related experiences is shown in Table 2. The mean level of depressive symptoms was 12.9 (SD = 4.9), while the mean level of general self-efficacy was 14.7 (SD = 3.1). Thirty-three percent of the sample reported to use social media daily for more than 3 h. Twenty-two percent had experienced being bullied on the internet, while 30% had experienced pressure toward having followers and receiving likes on social media. There were fewer participants with valid responses to the questions about having experienced social media-related conflicts or arguments in the family, or not getting enough sleep due to social media use. Among those with valid responses on these questions, 13% reported to have experienced social media-related conflicts or arguments in the family, while 49.5% reported to have experienced not getting enough sleep due to social media use.

**Table 2:** Depressive symptoms, psychological resources, social media use, and negative social media-related experiences in the sample.

**Characteristics**	***n***	**Values**
**Depressive symptoms**		**M (SD)**
Scale rating	126,445	12.9 (4.9)
**Psychological resources**		**M (SD)**
General self-efficacy	54,371	14.7 (3.1)
**Daily time on social media**	**n**	**%**
No time	3,359	2.4
< 30 min	12,967	9.3
30 min−1 h	17,593	12.6
1–2 h	24,602	17.6
2–3 h	24,522	17.5
>3 h	46,166	33.0
**Negative social media-related experiences**		
**Having been bullied on the**	**n**	**%**
**internet**		
Yes	30,129	21.5
No	104,048	74.4
**Arguments or conflict in the family**	**n**	**%**
Yes	4,986	3.6
No	33,465	23.9
**Not enough sleep**		
Yes	19,063	13.6
No	19,450	13.9
**Pressure to have followers and likes on SM**		
Yes	43,062	30.8
No	84,971	66.4

SM is social media. On all mandatory parts of the questionnaire, missing values represented < 10% of the possible responses: depressive symptoms 9.6%, daily time on social media 7.6%, bullied on the internet 4.1%, pressure to have followers and likes on social media 8.4%.

### Depressive symptom levels in sample subgroups

The levels of depressive symptoms in sample subgroups are shown in Table 3. Depressive symptom levels were higher in the higher age groups, and girls had higher levels than boys. Depressive symptoms were also higher among adolescents living in urban districts, compared to rural, and higher among those whose parents did not have higher education. Those with higher levels (i.e., above median levels) of general self-efficacy had lower levels of depressive symptoms, compared to their counterparts. Those who spent more time on social media reported higher levels of depressive symptoms, compared to those who used social media less. For all types of negative social media-related experiences, the levels of depressive symptoms were significantly higher among those who had experienced the event, compared to their counterparts.

**Table 3:** Depressive symptoms in sample subgroups.

**Subgroups**	**Depressive symptoms**		
**Age cohort**	**n**	**M (SD)**	**p**
8^th^ grade secondary school	24,477	11.9 (4.8)	< 0.001
9^th^ grade secondary school	24,487	12.6 (4.9)	
10^th^ grade secondary school	24,203	13.1 (4.9)	
1^st^ grade high school	20,602	13.1 (4.8)	
2^nd^ grade high school	17,240	13.4 (4.8)	
3^rd^ grade high school	11,601	13.9 (4.7)	
**Gender**		**M (SD)**	**p**
Boys	60,270	11.2 (4.3)	< 0.001
Girls	63,673	14.4 (4.8)	
**Centrality index**		**M (SD)**	**p**
1 (most central)	16,579	13.2 (4.9)	< 0.001
2	26,801	13.0 (4.9)	
3	34,186	13.0 (4.9)	
4	26,582	12.7 (4.8)	
5	16,823	12.5 (4.8)	
6 (least central)	5,386	12.5 (4.9)	
**Parents with higher**		**M (SD)**	**p**
**education**			
No parents with higher education	18,286	13.4 (5.2)	< 0.001
One parent with higher education	38,200	13.1 (4.9)	
Both parents with higher education	62,462	12.6 (4.7)	
**Psychological resources**		**M (SD)**	**p**
High GSE-5 (>Md)	18,009	11.0 (4.3)	< 0.001
Low GSE-5 (≤ Md)	35,263	13.8 (4.8)	
**Daily time on social media**		**M (SD)**	**p**
No time	3,114	11.3 (5.3)	< 0.001
< 30 min	12,325	11.2 (4.6)	
30 min−1 h	16,951	11.6 (4.4)	
1–2 h	23,807	12.2 (4.5)	
2–3 h	23,687	12.9 (4.6)	
>3 h	44,386	14.3 (5.0)	
**Negative social media-**			
**related experiences**			
**Experienced online**		**M (SD)**	**p**
**bullying**			
Yes	27,944	15.3 (4.9)	< 0.001
No	97,955	12.2 (4.6)	
**Arguments or conflict in**		**M (SD)**	**p**
**the family**			
Yes	4,839	15.3 (4.9)	< 0.001
No	32,604	12.5 (4.7)	
**Not enough sleep**		**M (SD)**	**p**
Yes	18576	14.2 (4.6)	< 0.001
No	18,927	11.5 (4.6)	
**Pressure to have followers**		**M (SD)**	**p**
**and likes**			
Yes	41,730	15.1 (4.6)	< 0.001
No	81,931	11.8 (4.6)	

GSE is General Self-Efficacy Scale.

In the pairwise comparisons for variables with three or more categories (data not shown), differences in depressive symptom levels between age groups were statistically significant with one exception (10^th^ grade vs. 1^st^ grade high school). Depressive symptom levels were also significantly different between levels of centrality, with two exceptions (between levels 2 and 3, and between levels 5 and 6). The highest levels of depressive symptoms were found among adolescents where none of the parents had higher education, whereas the lowest levels were found among adolescents where both parents had higher education. Higher levels of social media use were, for the most part, significantly related to higher levels of depressive symptoms, but depressive symptom levels were not significantly different between adolescents who did not use social media and those using social media for < 1 h daily.

### Social media use, negative social media-related experiences, and general self-efficacy associated with depressive symptoms

In preparation of the regression analyses, time spent on social media was re-categorized in order to obtain similar group sizes and a linear relationship with depressive symptoms (see Table 3). The two categories indicating the least daily time spent on social media were collapsed into one category (no social media use, up to 30 min daily). Otherwise, categories remained as before. No multicollinearity problems were detected (all VIFs ≤ 1.25, tolerance ≥ 0.80).

The results of the analysis of factors associated with depressive symptom levels are shown in Table 4. Crude associations with depressive symptoms were found for all included variables. More time spent daily on social media was associated with higher depressive symptom levels (β = 0.23, *p* < 0.001), as was experience with any of the negative social media-related events (β ranging between 0.20 and 0.32, all *p* < 0.001). Higher levels of general self-efficacy were associated with lower levels of depressive symptoms (β = −0.38, *p* < 0.001).

**Table 4:** Linear regression analysis showing associations between daily time spent on social media, negative social media-related experiences, general self-efficacy, and depressive symptoms.

**Independent variables**	**Model 0**	**Model 1** **(*n* = 36,961)**	**Model 2** **(*n* = 26,865)**	**Model 3** **(*n* = 24,474)**
**Social media use**	β	β	β	β
Daily time spent on social media	0.23	0.09	0.08	0.04
**Explained variance (Adjusted R** ^**2**^ **)**		**5.2%**	**4.9%**	**4.9%**
**Negative social media-related experiences**				
Experienced online bullying	0.27	0.21	0.17	0.19
Arguments or conflict in the family	0.20	0.09	0.06	0.06
Not enough sleep	0.28	0.17	0.15	0.12
Pressure toward having followers and likes	0.32	0.22	0.19	0.13
**Explained variance (Adjusted R** ^**2**^ **)**		**21.2%**	**20.4%**	**20.1%**
**Psychological resources**				
General self-efficacy	−0.38	-	−0.29	−0.26
**Explained variance (Adjusted R** ^**2**^ **)**			**28.3%**	**27.7%**
**Sociodemographic variables**				
Higher age	0.11	-	-	0.11
Female gender	0.33	-	-	0.18
Centrality	−0.05	-	-	−0.03
Parents with higher education	−0.06	-	-	−0.03
**Explained variance (Adjusted R** ^**2**^ **)**				**31.8%**

Dependent variable is depressive symptom level. Higher values on “centrality” indicates more rural. Table content is standardized β values. All values of β and R^2^ are statistically significant at *p* < 0.001. Model 0 is single regression coefficients without any adjustment (crude associations). In Model 1, time on social media and social media-related experiences are entered in two separate blocks. Model 2 is Model 1 with the addition of general self-efficacy. Model 3 is Model 2 adjusted by sociodemographic variables. Bold is used to separate explained variance from beta values.

As shown for the multivariate Model 1, more time spent on social media was associated with higher depressive symptom levels, but the association was weaker compared with the crude association (β = 0.09, *p* < 0.001). Having experienced any of the negative social media-related events was associated with higher depressive symptom levels, with associations being of varying strength (β ranging between 0.09 and 0.22, all *p* < 0.001). Variation in the negative social media-related events accounted for a larger proportion of the outcome variance (16.0%), compared to the variation in time spent on social media (5.2%).

When included in Model 2, higher self-efficacy was related to lower levels of depressive symptoms (β = −0.29, *p* < 0.001), explaining and additional 7.9% of the variance in depressive symptoms. The inclusion of general self-efficacy did not substantially change the associations between social media use, negative social media-related experiences, and depressive symptoms.

After adjusting by sociodemographic variables in Model 3, time spent daily on social media and having experienced any of the negative social media-related events were still associated with higher depressive symptom levels, and higher general self-efficacy was still related to lower depressive symptom levels. Higher age, female gender, living in more central areas, and having parents who did not have higher education were associated with higher depressive symptom levels.

### Interaction analyses

Interaction analyses were performed to examine whether associations between negative social media experiences and depressive symptoms were moderated by gender and levels of general self-efficacy, respectively. Interactions were added in two subsequent iterations of the Model 3 regression analysis. In the first iteration, interaction terms gender × negative social media-related experiences (i.e., four separate interaction terms) were added to the Model 3 predictors, and in the second we added the interaction terms general self-efficacy × negative social media-related experiences. Gender significantly interacted with “arguments or conflict in the family” (*p* < 0.001), whereas the remaining three interaction terms yielded non-significant results. General self-efficacy interacted with “arguments or conflict in the family” and “pressure toward having followers and likes” (both *p* < 0.001), whereas the two remaining interaction terms were non-significant. Based on these results, we repeated the Model 3 regression analysis separately for boys and girls, and for participants with higher and lower general self-efficacy scores (spilt by the median score).

The results for boys and girls are displayed in Table 5. The pattern of associations was mostly identical for the two genders. However, having experienced conflicts or arguments related to social media use was associated with a higher level of depressive symptoms among girls, but not among boys. Also, for girls the model accounted for a somewhat larger proportion of the variance in depressive symptoms, and the association between higher general self-efficacy and lower levels of depressive symptoms was stronger than for boys.

**Table 5:** Linear regression analysis showing associations between daily time spent on social media, negative social media-related experiences, general self-efficacy, and depressive symptoms by gender.

**Independent variables**	**Boys** **(*n* = 11,574)**	**Girls** **(*n* = 12,900)**
**Social media use**	β	β
Daily time spent on social media	0.02	0.05^***^
**Explained variance** **(Adjusted R**^**2**^**)**	**1.3%** ^***^	**2.6%** ^***^
**Negative social media-**		
**related experiences**		
Experienced online bullying	0.22^***^	0.19^***^
Arguments or conflict in the family	0.02	0.09^***^
Not enough sleep	0.15^***^	0.11^***^
Pressure toward having followers and likes	0.13^***^	0.13^***^
**Explained variance** **(Adjusted R**^**2**^**)**	**14.4%** ^***^	**15.0%** ^***^
**Psychological resources**		
General self-efficacy	−0.22^***^	−0.32^***^
**Explained variance** **(Adjusted R**^**2**^**)**	**18.7%** ^***^	**24.5%** ^***^
**Sociodemographic**		
**variables**		
Higher age	0.12^***^	0.12^***^
Centrality	−0.04^***^	−0.02^**^
Parents with higher education	−0.03^**^	−0.03^***^
**Explained variance** **(Adjusted R**^**2**^**)**	**20.5%** ^***^	**26.1%** ^***^

Dependent variable is depressive symptom level. Higher values on “centrality” indicates more rural. Table content is standardized β values. The models show direct associations when all variables are included.

*** *p* < 0.001,

** *p* < 0.01. Bold is used to separate explained variance from beta values.

The results for higher and lower levels of general self-efficacy are displayed in Table 6. The pattern of associations was practically identical for the two groups, and the models accounted for similar proportions of the outcome variance. The association between female gender and depressive symptoms was slightly stronger for adolescents with lower levels of general self-efficacy, compared to those with higher levels.

**Table 6:** Linear regression analysis showing associations between daily time on social media, social media-related experiences, and depressive symptoms by general self-efficacy levels.

**Independent variables**	**Low self-efficacy** **(*n* = 15,892)**	**High self-efficacy** **(*n* = 8,582)**
**Social media use**	β	β
Daily time spent on social media	0.04	0.04
**Explained variance** **(Adjusted R**^**2**^**)**	**4.2%**	**3.9%**
**Social media-related**	β	β
**experiences**		
Experienced online bullying	0.22	0.20
Arguments or conflict in the family	0.08	0.08
Not enough sleep	0.12	0.16
Pressure toward having followers and likes	0.13	0.15
**Explained variance** **(Adjusted R**^**2**^**)**	**17.0%**	**18.2%**
**Sociodemographic**	β	β
**variables**		
Higher age	0.10	0.11
Female gender	0.23	0.16
Centrality	−0.03	−0.04
Parents with higher education	−0.03	−0.04
**Explained variance** **(Adjusted R**^**2**^**)**	**22.7%**	**21.9%**

Dependent variable is depressive symptom level. Higher values on “centrality” indicates more rural. Table content is standardized beta values. The models show direct associations when all variables are included. All values of β and R^2^ are statistically significant at *p* < 0.001. Bold is used to separate explained variance from beta values.

### Differences between the initial and the final sample

Given the loss of participants in the multivariate analysis, we compared the sample included in the Model 3 regression analysis with the initial sample. In comparison to the initial sample, the restricted sample included in the Model 3 analysis had a lower level of depressive symptoms (M = 12.8 vs. M = 12.9, Cohen's *d* = 0.02, *p* < 0.001) but a higher level of social media use (M = 4.6 vs. M = 4.5, Cohen's *d* = 0.09, *p* < 0.001). The restricted sample also had a higher proportion of participants in senior high school (54.3 vs. 37.8%), a higher proportion of girls (53 vs. 50%), a higher proportion had both parents without higher education (17.0 vs. 15.5%), and a higher proportion living in the less central areas of the country (44.3 vs. 36.9%, all *p* < 0.001).

## Discussion

### Main summary of results

This study found that negative social media-related experiences were associated with higher levels of depressive symptoms among Norwegian adolescents. When including negative social media-related experiences as independent variables together with daily time spent on social media, the association between time spent on social media and depressive symptoms was weakened, suggesting that negative social media-related events were responsible for some (but not all) of the association between time spent using social media and higher levels of depressive symptoms. Higher general self-efficacy levels were associated with lower levels of depressive symptoms.

### Factors associated with depressive symptoms

In this study, more time spent on social media use was associated with higher levels of depressive symptoms. This corresponds with results from previous reviews that have suggested social media use to be associated with higher levels of depressive symptoms in adolescence (53). However, using a wider lens, a recent review and meta-analysis reported that associations between more social media use and poorer mental health varied considerably, with effect sizes ranging between 0.02 (*ns*) and 0.17 (54). In view of the variable results between studies and the small effect sizes often obtained, other factors may influence the relationship between social media use and depressive symptoms in adolescents (55).

In this study, we found that the inclusion of negative social media-related experiences in the regression model weakened the initial association between social media use and depressive symptoms, hence possibly accounting for some of the covariation between social media use and depressive symptoms. Among the included negative experiences, having experienced online bullying was shown to be most strongly associated with depressive symptoms. A relationship between having experienced online bullying and higher levels of depressive symptoms is consistent with findings from previous studies (56). These results support the idea that the content of social media use–what people experience during, and as a consequence of, social media use–is important to consider in relation to mental health outcomes, and that time spent on social media is merely one aspect of the social media exposure. While the results indicate that negative experiences related to social media use are relevant to consider, they also support the notion that there remains an independent relationship between time spent on social media and depressive symptoms in adolescents. This remaining relationship may be bi-directional or reversed, as recently suggested (57).

Almost half of the participants who responded to the questions related to sleep and social media use, reported not getting enough sleep due to social media use. This is in accordance with a recent Norwegian research report, stating that 50% of adolescents go to bed later than they should due to social media use (8). Previous studies have also suggested that social media use may cause sleep problems (21, 22), and our study substantiated higher depressive symptom levels among adolescents who have experienced sleep problems due to social media use. In addition, the results from our study showed that other problematic social media related experiences, such as “conflicts or arguments in the family” and “pressure to have many followers and likes,” were associated with higher depressive symptom levels. We should note that family conflicts related to social media hinges not only on the adolescent's social media use, but also on parents' attitudes and behaviors toward their adolescent's use of social media. Studies have shown that over the past decades parents have become increasingly aware of, and involved with, their children's social media use (58, 59), which in turn may lead to more family conflict centered around the issue (12). In combination, the findings suggest that substantial proportions of adolescents experience problematic consequences of their social media use, and that having such experiences are related to higher depressive symptoms. However, the nature of the relationship is uncertain, and bi-directional relationships are viable (57).

Moreover, the frequency of experiencing negative social media-related events may differ between groups. For example, adolescents from less affluent backgrounds have been found to report negative experiences from social media use more often than those from more affluent backgrounds (9), and possibly, associations between social media-related experiences and depressive symptoms may differ between groups with higher or lower vulnerability. Supporting this view, a recent study concluded that getting fewer likes on social media was a greater risk factor for negative affect and thoughts and feeling rejected among adolescents who were already victims of school bullying, compared to those who were not (19).

While this study has focused on depressive symptoms as the potential outcome of social media use and -encounters, social media may also have a beneficial effect on adolescent wellbeing. Social media may ease the access to supports and allow for a diversity of interactions and friendships (53), and for some, the virtual interaction may be easier than face-to-face social interaction (14–16). In a Norwegian qualitative study adolescents revealed that social media contributes to amplify social relations, as it is much easier to communicate and keep in touch *via* social media than in real life (60). However, negative experiences on social media have been found to be more potent than positive experiences, as negative experiences have been significantly associated with higher levels of depressive symptoms, while positive experiences have been associated with minor and not statistically significant decreases in depressive symptoms (61). Individuals with depressive symptoms may also be more inclined to engage in social media for various reasons (57) and they may experience more negative interactions while doing so (61).

In this study, higher general self-efficacy levels were significantly associated with lower levels of depressive symptoms. Similarly, a previous study also showed that self-efficacy was related to depressive symptom levels, both concurrently and prospectively, but the nature of the associations varied by self-efficacy domain (62): a strong sense of efficacy for regulating negative affect was related to lower depressive symptoms, while stronger empathic self-efficacy was related to higher depressive symptom levels. In addition, a more recent study examining a cross-lagged model of associations found that depressive symptom levels predicted academic and emotional self-efficacy 6 months later, whereas self-efficacy did not predict subsequent depressive symptoms (63). Thus, the issue of causality in the relationship between self-efficacy and depressive symptoms is debated, and cyclical relationships are also viable. Previous studies have found that self-efficacy moderated associations between social media use and mental health (36, 37). Similarly, our study showed that general self-efficacy significantly moderated two of the associations between negative social media-related experiences and depressive symptoms. However, associations were only marginally different between adolescents with higher and lower levels of self-efficacy. In addition, including general self-efficacy in the regression model (Model 3) did not substantially change the associations between social media use, negative social media-related experiences, and depressive symptoms. Thus, despite significant interaction effects, the comparison of effect sizes in our study supports the view that associations between social media use and negative social media-related experiences and depressive symptoms do not differ substantially between adolescents with different levels of self-efficacy.

Comparisons with other studies are, to some extent, hampered by different questions and conceptualizations used across studies. For example, 22 % of the adolescents in this study reported to have experienced online bullying. This proportion is higher compared to the results of Craig et al. (20), where 12% reported having experienced cyberbullying on social media. However, these results are not fully comparable, given that more adolescents would logically report having experienced online bullying (as we asked in this study), compared to those having experienced cyberbullying on social media (as asked by Craig and co-workers). Online bullying, the concept used in our study, would logically encompass bullying within a wider internet-based context, including e.g., online gaming, and is not necessarily restricted to the social media context. For these reasons, our results should be interpreted with caution.

### Study strengths and limitations

A considerable strength of the present study is the high participation rate, the large sample size, and the use of recently collected data (2021) which provide updated descriptions of adolescents' perceptions of life in Norway. The very large sample, and the resulting high statistical power, give rise to challenges in the interpretation of associations, where statistically significant results must be scrutinized with an emphasis on effect size. In this study, for example, we found several statistically significant interaction effects, yet close to negligible group differences when comparing categories of the moderator variables.

Another limitation is that the sample used for the Model 3 regression analysis was different from the initial sample with regards to the participants' level of depressive symptoms, time spent on social media use, age, gender, centrality, and their parents' educational level. Thus, our results concerned with factors associated with depressive symptoms are not fully representative of the general population of adolescents, in particular with regards to the age distribution.

A limitation of this study is the cross-sectional design, which precludes inferences about causal relationships. Depressive symptoms may not only be a result of social media use and exposure; such symptoms may also increase social media use and potentially the risk of experiencing adverse events, such as online bullying. In addition, the study relies on self-report measures, some of which with unknown psychometric properties, which may have led to unidentified misclassifications or measurement errors. While the questions used to assess negative social media-related experiences have been used in the Ungdata surveys for several years (64), we have no information about the validity of the questions. Furthermore, the associations found between social media use and depressive symptoms might be influenced by other factors that were not controlled for in the study.

## Conclusion and implications for future research

This study showed that negative social media-related events were associated with higher levels of depressive symptoms among adolescents in Norway, and negative social media-related events appeared to account for some of the association between time spent using social media and higher levels of depressive symptoms. Higher general self-efficacy levels were associated with lower levels of depressive symptoms. More knowledge is needed about what types of events might constitute both positive and negative events related to social media use, and what outcomes they might produce. In particular, further research on cyberbullying and its contexts, is needed. Such research may include a larger diversity of online platforms where people interact with each other, including both social network platforms and online gaming platforms. Future research may also focus on identifying potential moderating variables, to enable a more comprehensive understanding of how social media use and -exposure may be differently associated with mental health outcomes in various groups of adolescents.

## Data availability statement

The data analyzed in this study is subject to the following licenses/restrictions: The data and materials from the Ungdata Surveys are stored in a national database administered by NOVA. The data are available for research purposes upon application. Further information about the study and the questionnaires can be found on the web page https://www.nsd.no/nsddata/serier/ungdata_eng.html. Requests to access these datasets should be directed to ungdata@oslomet.no.

## Ethics statement

The studies involving human participants were reviewed and approved by Institutional Ethics Review Board, Inland Norway University of Applied Sciences. Written informed consent from the participants' legal guardian/next of kin was not required to participate in this study in accordance with the national legislation and the institutional requirements.

## Author contributions

TB analyzed the data and drafted the manuscript. AS, TS, AK, LL, and ML provided input to the analysis and presentation and edited the manuscript. All authors agreed to be responsible for the final submitted version of the manuscript.
